# Supplementary material for: Psychological consequences of long COVID: comparing trajectories of depressive and anxiety symptoms before and after contracting SARS-CoV-2 between matched long- and short-COVID groups
Source: Br J Psychiatry. 2023 Feb;222(2):74–81. doi: 10.1192/bjp.2022.155 (PMC7614126; doi:10.1192/bjp.2022.155)
Supplement: Supplementary file 1 [file S0007125022001556sup001.docx]

Supplementary Material

Model specification:

$Y_{ij}=\pi_{0i}+\pi_{1i}{time}_{ij} + \pi_{2i}{time}_{ij}^{2} +\varepsilon_{ij}$ (Eq. 1)

$\pi_{0i}=\gamma_{00}+\gamma_{01}{group}_{i}+\gamma_{01}{month}_{i}+\zeta_{0i}$ (Eq. 2)

$\pi_{1i}=\gamma_{10}+\gamma_{11}{group}_{i}+\zeta_{1i}$ (Eq. 3)

$\pi_{2i}=\gamma_{20}+\gamma_{21}{group}_{i}+\zeta_{2i}$ (Eq. 4)

The growth curve model is presented mathematically in Equation (1) to (4). Equation (1) addresses intra-individual growth where $Y_{ij}$, depressive or anxiety symptoms for the individual i at time j, is a function of time and its quadratic term. $\pi_{0i}$ represents the individual i’s initial status. $\pi_{1i}$ and $\pi_{2i}$ represent growth rates and $\varepsilon_{ij}$ is the residual term. Equation (2) to (4) addresses inter-individual differences in the initial status (intercept) and growth rates. $\gamma_{00}$, $\gamma_{10}$and $\gamma_{20}$ represent the population average initial status (intercept) and growth rates. $\zeta_{0i}$, $\zeta_{1i}$ and $\zeta_{2i}$ are parameter residuals.


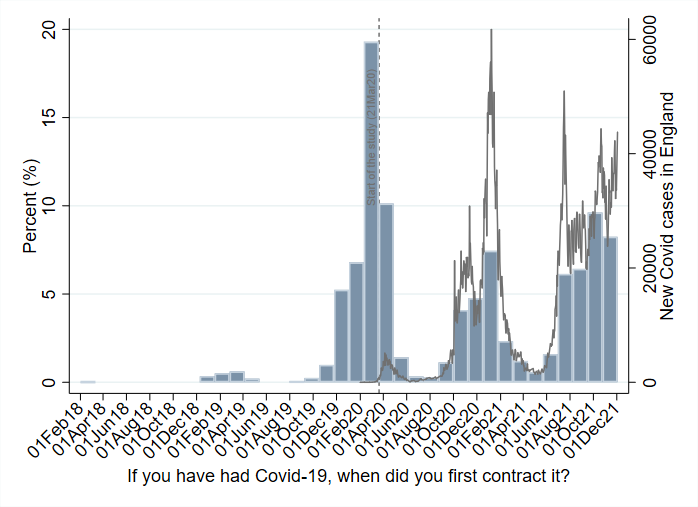


Fig S1. The date of first contract (N=4,938) compared to the national statistics of COVID-19 cases in England

Sources: COVID-19 Social Study, https://coronavirus.data.gov.uk/

Other countries in the UK (18%)

Not completed Covid-19 module in Nov 2021 (58%)

Missing data general (N=11%)

Missing data on Covid-19 (1%)

Not had COVID-19 by Nov 2021 (N=88%)

First contract before 21 Mar 2020 (28%)

*See Fig S2

Covid-19 sample

(N=3 211)

Covid-19 sample

(N=3 115)

Had Covid-19 more than once (3%)

Total sample size

(N=73 242)

Living in England

(N=59 803)

Completed the Covid-19 module in Nov 2021

(N=25 289)

Analytical sample

(N=22 528)

Covid-19 sample

(N=4 938)

Covid-19 sample

(N=4 887)

Fig S2. Sample selection process

Fig S3. (a) Distribution of survey months since contracting COVID-19 in the one-to-one matched sample (total number of observations=13,325), (b) Distribution of the gap (day) between self-reported COVID-19 date and the closest survey date (either before or after, number of participants=962)


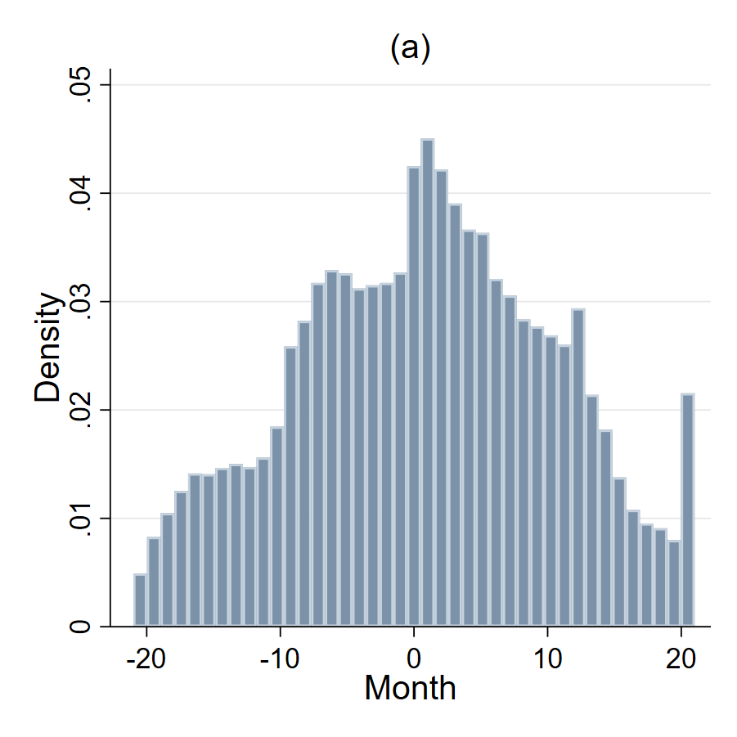

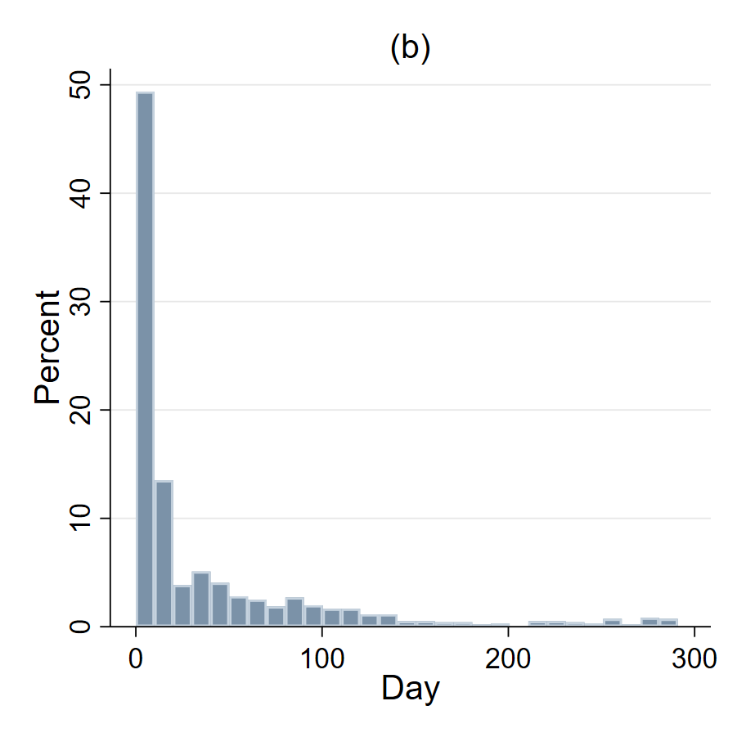


Fig S4. Propensity score density before and after matching


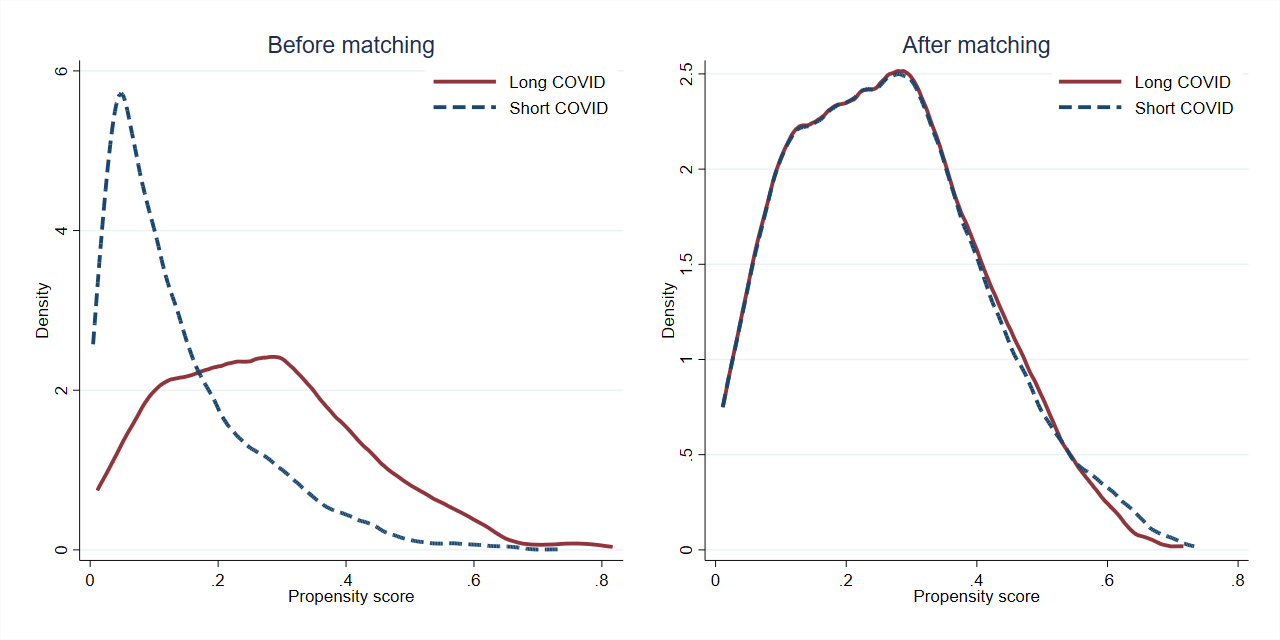


Fig S5. Standardised percentage bias for each covariate before and after matching


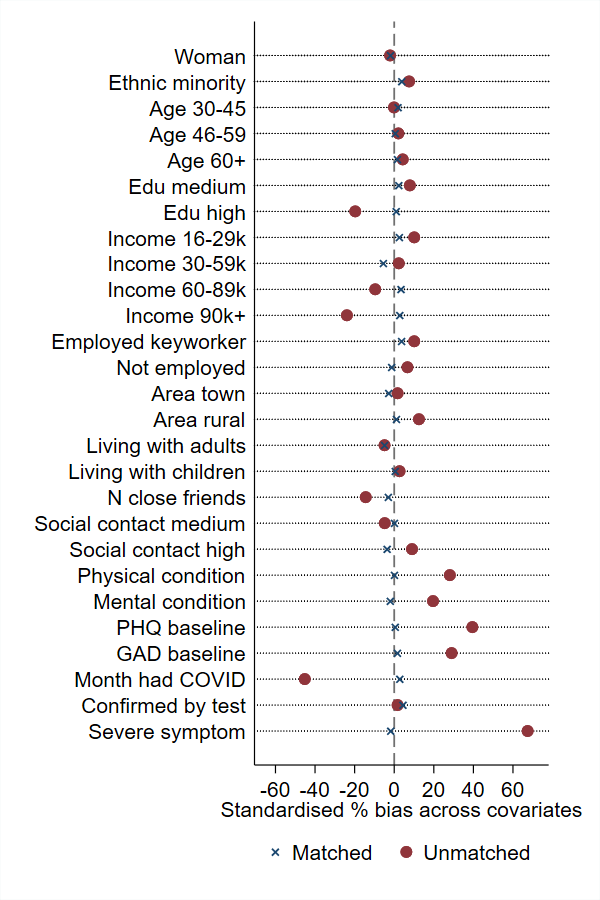


Fig S6. Predicted growth trajectories by Covid-19 group with 95% confidence intervals


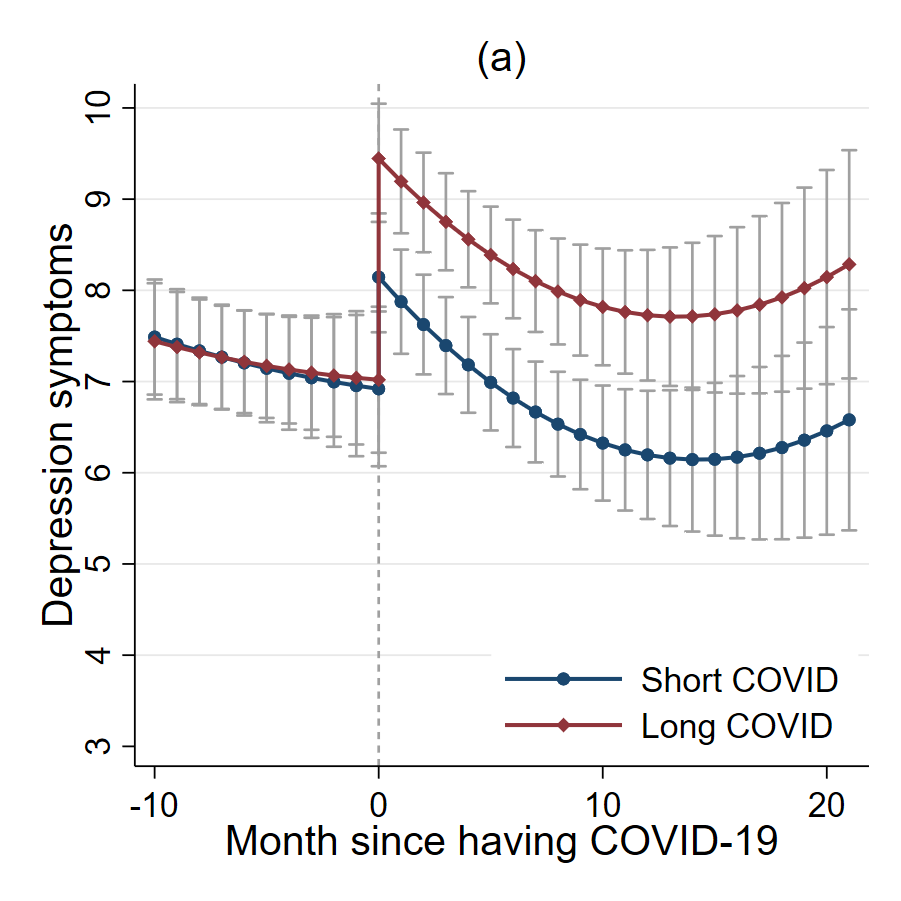

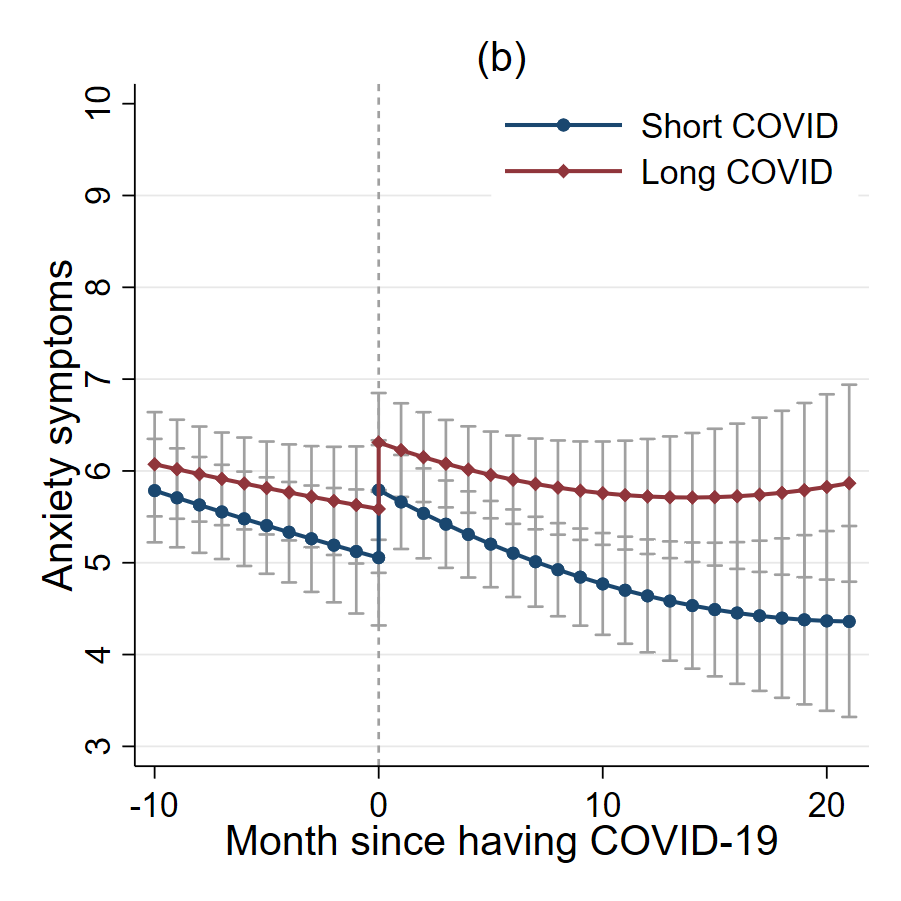


Fig S7. Propensity score density before and after matching based on one-to-many nearest neighbour matching


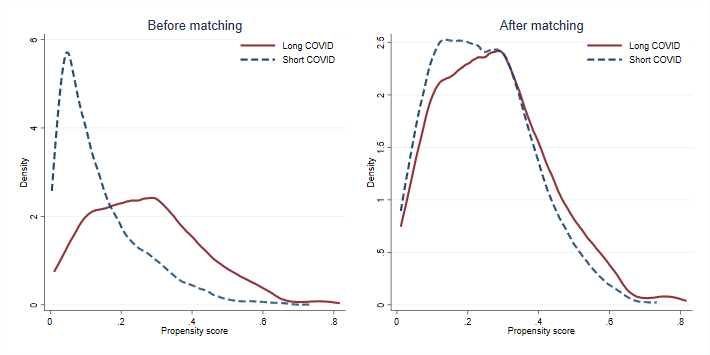


Fig S8. Predicted growth trajectories by Covid-19 group with 95% confidence intervals based on one-to-many nearest neighbour matching


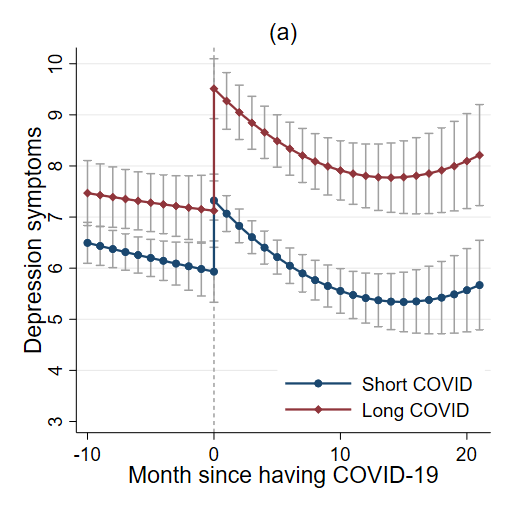

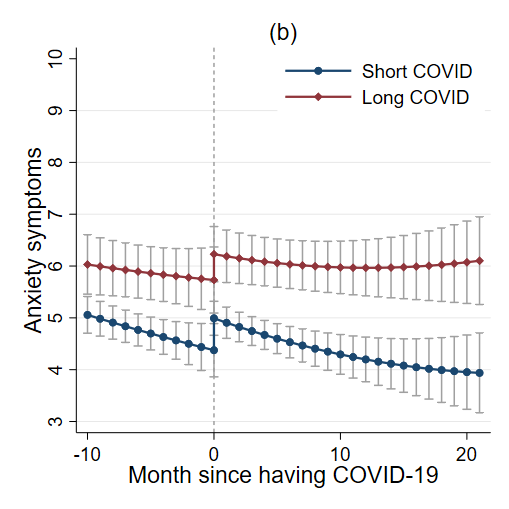


Fig S9. Predicted growth trajectories by Covid-19 group with 95% confidence intervals based on kernel matching *(NB these sensitivity analyses yielded less balanced matches so apparent differences in pre-infection mental health should be interpreted with caution)*


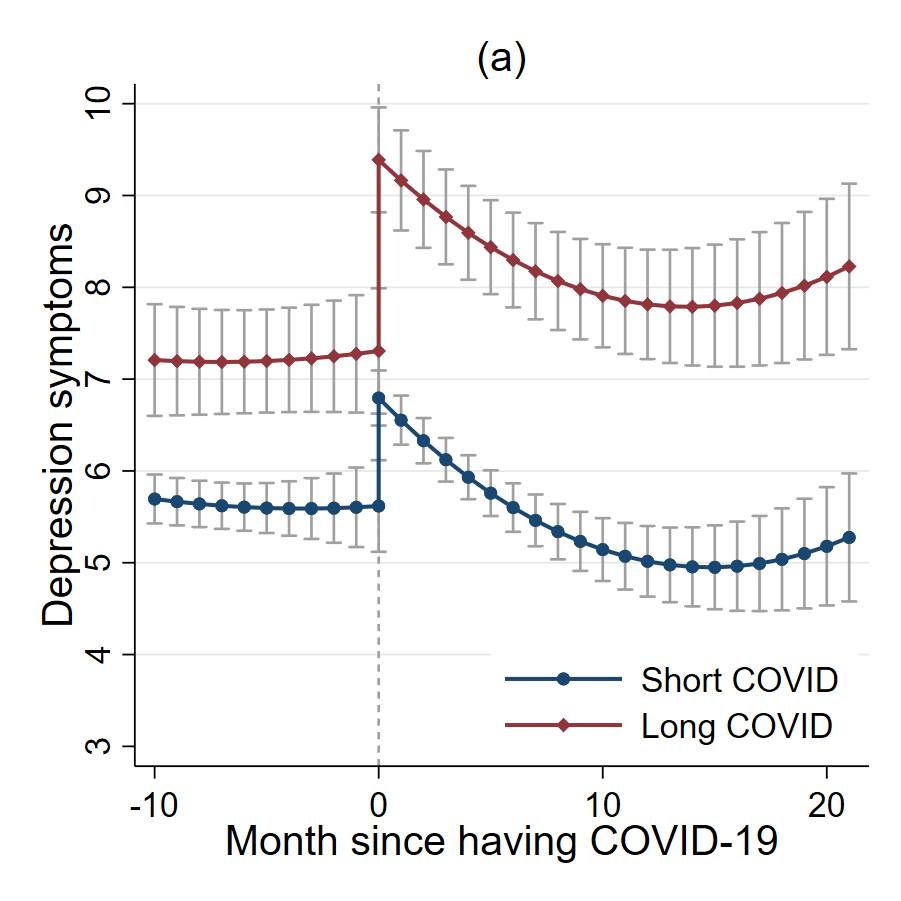

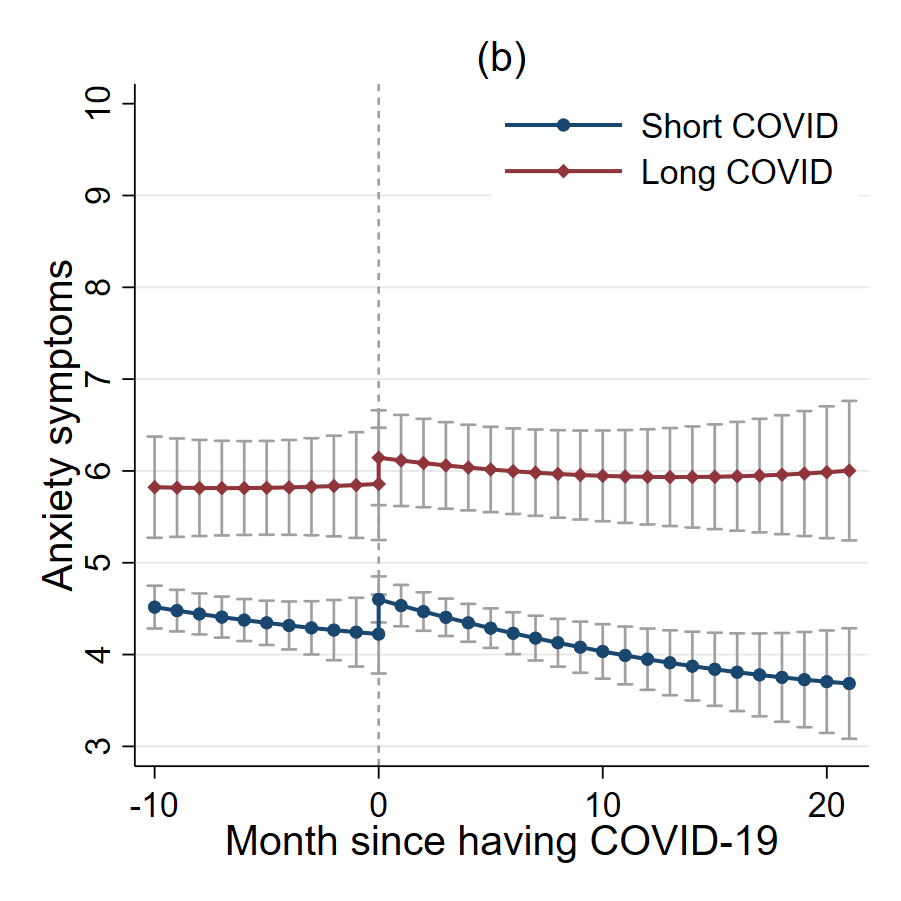


Fig S10. Predicted growth trajectories by Covid-19 group with 95% confidence intervals based on one-to-one matching excluding cases based on symptoms


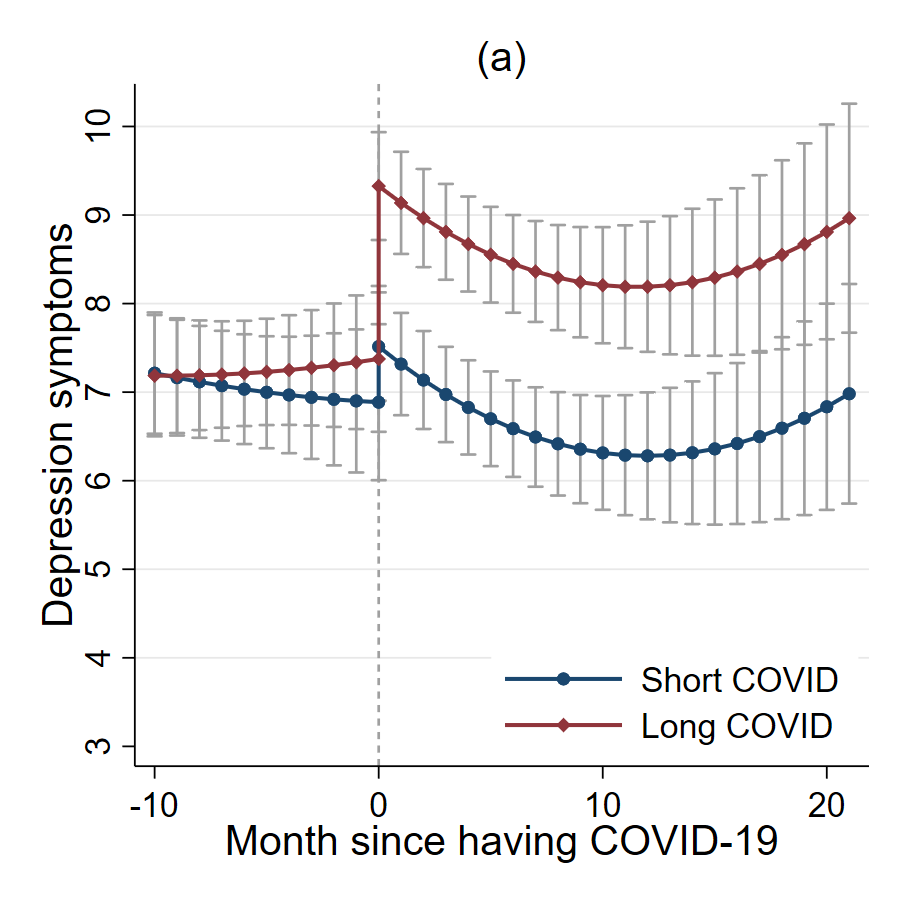

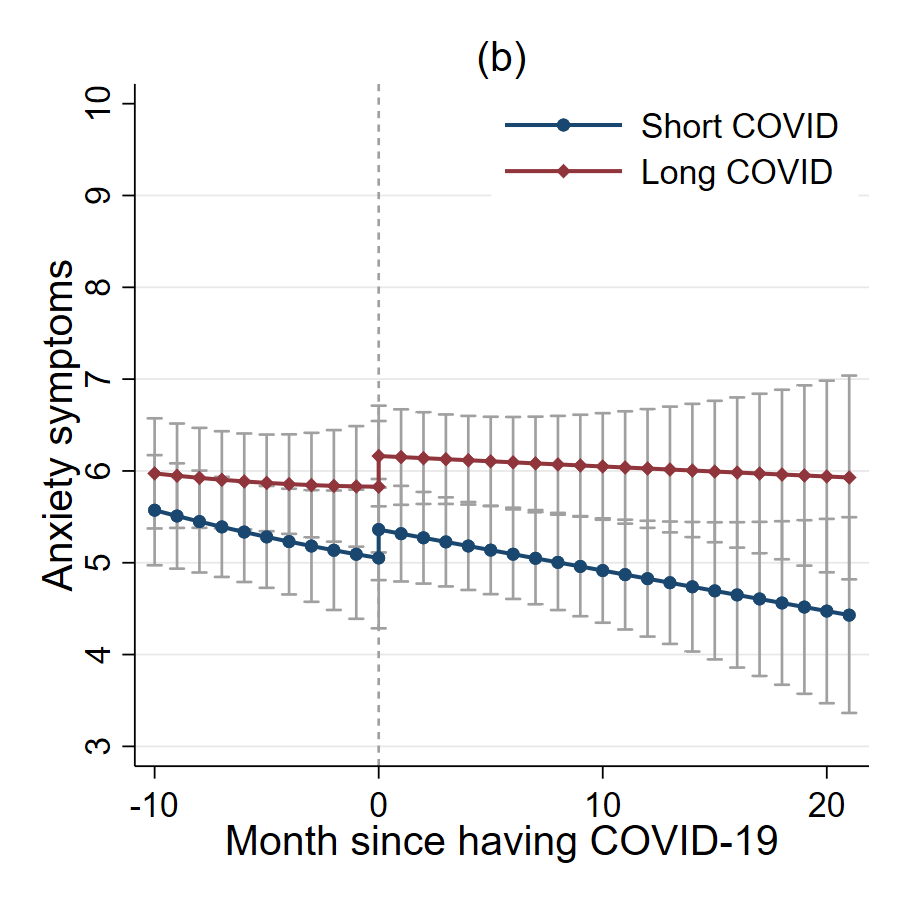


Fig S11. Predicted growth trajectories by Covid-19 group with 95% confidence intervals based on one-to-one matching excluding people who were unsure about their long Covid diagnosis


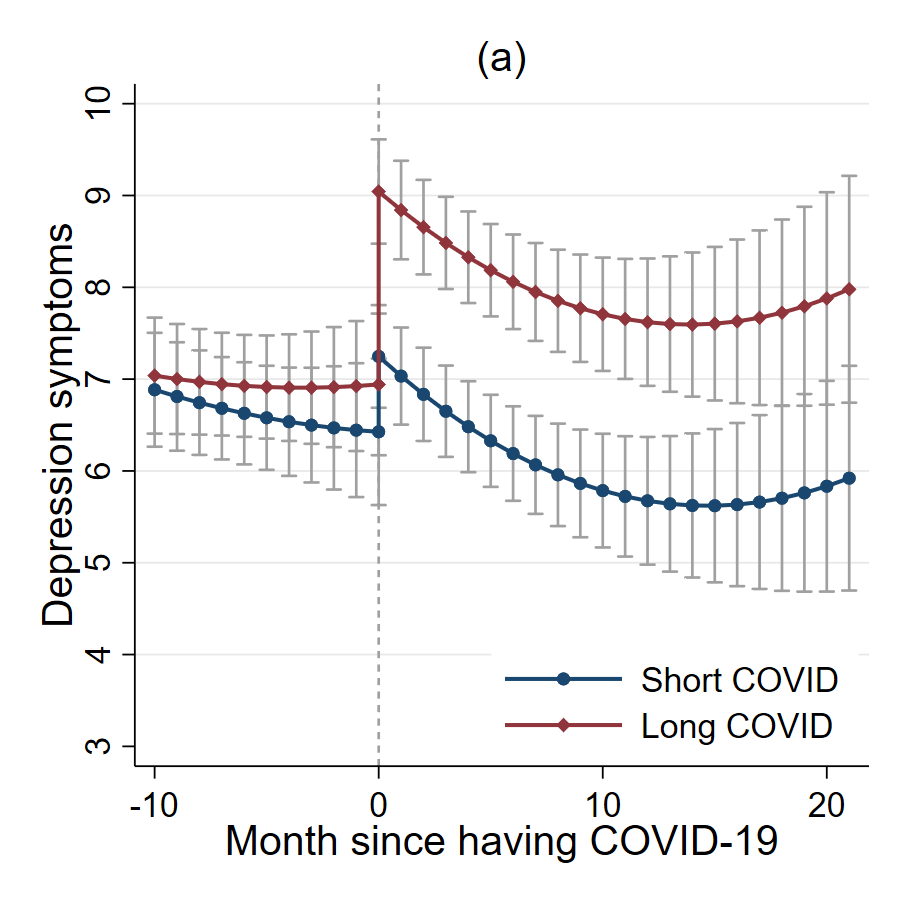

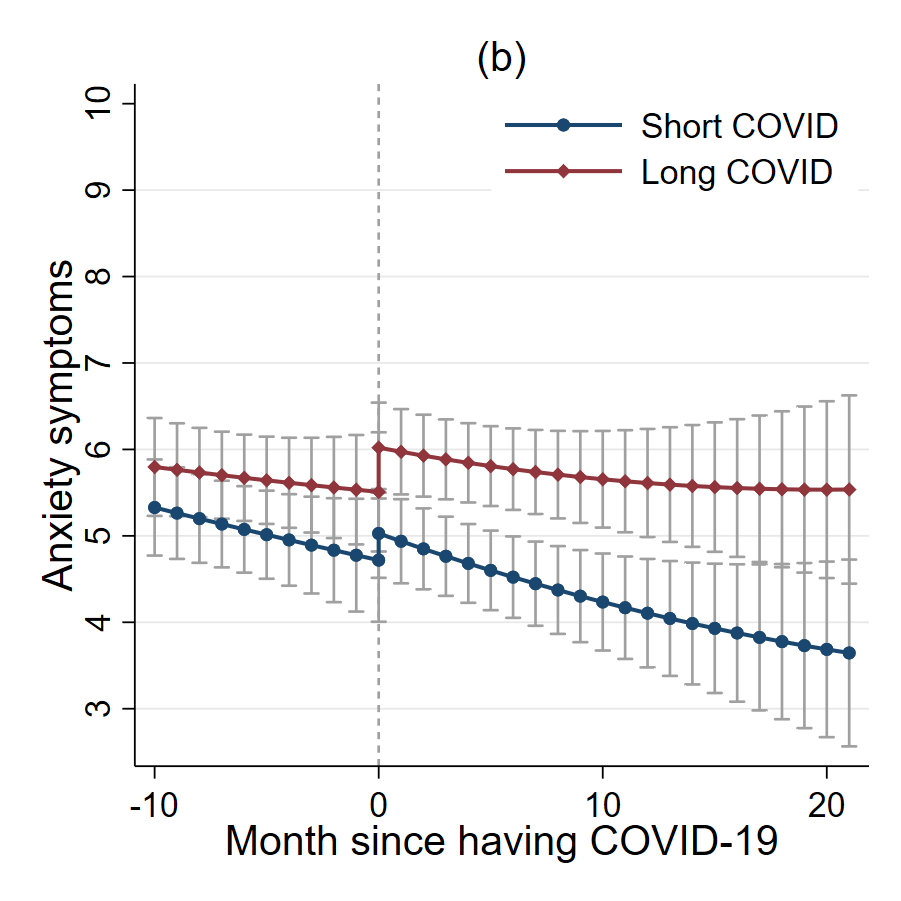


Table S1 Results from Propensity Score Matching (one-to-one matching without replacement)

|  | Coef. | SE | 95% CI | p |
| --- | --- | --- | --- | --- |
| Women (vs men) | -0.33 | 0.13 | [-0.60--0.07] | 0.013 |
| Ethnic minority (vs white) | 0.30 | 0.23 | [-0.16-0.76] | 0.197 |
| Age 30-45 (vs 18-29) | 0.48 | 0.24 | [0.01-0.95] | 0.046 |
| Age 46-59 (vs 18-29) | 0.45 | 0.24 | [-0.01-0.91] | 0.056 |
| Age 60+ (vs 18-29) | 0.64 | 0.26 | [0.13-1.14] | 0.013 |
| Education: A levels or equivalent (vs GCSE or below) | -0.23 | 0.19 | [-0.60-0.13] | 0.208 |
| Education: Degree or above (vs GCSE or below) | -0.40 | 0.16 | [-0.72--0.09] | 0.013 |
| Income: ≤£16,000-29,999 (vs <£16,000) | -0.18 | 0.19 | [-0.55-0.19] | 0.334 |
| Income: ≤£30,000-59,999 (vs <£16,000) | -0.25 | 0.19 | [-0.61-0.12] | 0.187 |
| Income: ≤£60,000-89,999 (vs <£16,000) | -0.48 | 0.22 | [-0.91--0.05] | 0.029 |
| Income: ≥£90,000 (vs <£16,000) | -0.93 | 0.26 | [-1.43--0.42] | <0.001 |
| Employed, non-keyworker (vs Employed, keyworker) | 0.14 | 0.13 | [-0.11-0.39] | 0.282 |
| Not employed (vs Employed, keyworker) | -0.03 | 0.15 | [-0.32-0.27] | 0.864 |
| Area of living: Town (vs City) | 0.09 | 0.12 | [-0.15-0.34] | 0.452 |
| Area of living: Rural (vs City) | 0.51 | 0.15 | [0.21-0.81] | 0.001 |
| Living with adult only (vs Living alone) | 0.13 | 0.17 | [-0.2-0.46] | 0.444 |
| Living with children (vs Living alone) | 0.39 | 0.18 | [0.03-0.74] | 0.032 |
| Number of close friends | -0.01 | 0.02 | [-0.05-0.03] | 0.655 |
| Social contact: Once or twice a week (vs Twice a month or less) | -0.08 | 0.13 | [-0.33-0.18] | 0.553 |
| Social contact: Three times a week or more (vs Twice a month or less) | -0.05 | 0.14 | [-0.33-0.23] | 0.733 |
| Physical health condition: Yes (vs No) | 0.32 | 0.11 | [0.10-0.54] | 0.004 |
| Mental health condition Yes (vs No) | 0.01 | 0.15 | [-0.28-0.29] | 0.955 |
| Baseline depressive symptoms | 0.05 | 0.02 | [0.02-0.08] | 0.002 |
| Baseline anxiety symptoms | 0.00 | 0.02 | [-0.03-0.04] | 0.885 |
| Month had COVID-19 | -0.09 | 0.01 | [-0.11--0.07] | <0.001 |
| Confirmed by testing (COVID-19 or antibody test): Yes (vs No) | 0.69 | 0.14 | [0.41-0.98] | <0.001 |
| Severe symptoms (hospitalised or had to stay in bed): Yes (vs No) | 1.37 | 0.13 | [1.12-1.63] | <0.001 |
| Constant | -2.47 | 0.41 | [-3.28--1.66] | <0.001 |

Table S2 Sample characteristics by matched Covid-19 group

|  |  | Long Covid  (N=481) | Short Covid  (N=481) |
| --- | --- | --- | --- |
| Gender | Men | 21.8% | 21.0% |
|  | Women | 78.2% | 79.0% |
| Ethnicity | White | 94.2% | 95.0% |
|  | Ethnic minority | 5.8% | 5.0% |
| Age group | 18-29 | 6.0% | 7.7% |
|  | 30-45 | 31.6% | 30.8% |
|  | 46-59 | 39.1% | 38.9% |
|  | 60+ | 23.3% | 22.7% |
| Education | GCSE or below | 15.4% | 16.6% |
|  | A levels or equivalent | 19.5% | 18.7% |
|  | Degree or above | 65.1% | 64.7% |
| Annual income | <£16,000 | 14.3% | 14.8% |
|  | ≤£16,000-29,999 | 23.7% | 22.7% |
|  | ≤£30,000-59,999 | 38.3% | 41.0% |
|  | ≤£60,000-89,999 | 16.2% | 15.0% |
|  | ≥£90,000 | 7.5% | 6.7% |
| Employment status | Employed, keyworker | 39.1% | 40.1% |
|  | Employed, non-keyworker | 32.6% | 31.0% |
|  | Not employed | 28.3% | 28.9% |
| Area of living | City | 32.0% | 31.0% |
|  | Town | 46.8% | 48.2% |
|  | Rural | 21.2% | 20.8% |
| Living status | Living alone | 14.8% | 12.5% |
|  | Living with adult only | 48.2% | 50.7% |
|  | Living with children | 37.0% | 36.8% |
| Social contact | Twice a month or less | 35.3% | 33.7% |
|  | Once or twice a week | 33.3% | 33.3% |
|  | Three times a week or more | 31.4% | 33.1% |
| Number of close friends |  | 4.7 (3.1) | 4.8 (3.1) |
| Physical health condition | Yes | 44.1% | 44.1% |
|  | No | 55.9% | 55.9% |
| Mental health condition | Yes | 23.1% | 23.9% |
|  | No | 76.9% | 76.1% |
| Baseline depressive symptoms | | 8.3 (5.9) | 8.2 (6.0) |
| Baseline anxiety symptoms | | 6.5 (5.4) | 6.4 (5.2) |
| Month had COVID-19 |  | 10.5 (6.5) | 10.3 (7.1) |
| Confirmed by testing (COVID-19 or antibody test) | Yes | 74.6% | 72.8% |
|  | No | 25.4% | 27.2% |
| Severe symptoms (hospitalised or had to stay in bed) | Yes | 81.7% | 82.5% |
|  | No | 18.3% | 17.5% |

Table S3. Detailed Covid experience measures by matched Covid-19 group

|  |  | Long Covid | Short Covid | Total |
| --- | --- | --- | --- | --- |
| Test | Yes, a medical professional has formally diagnosed me with long Covid | 112  23.3% | -- | 112 11.6% |
|  | Yes, I have not been formally diagnosed but consider myself to have Long Covid | 369  76.7% | -- | 369 38.4% |
|  | No, I do not consider myself to have Long Covid | -- | 369  76.7% | 369 38.4% |
|  | I am unsure | -- | 112  23.3% | 112 11.6% |
|  | **Total** | **481** | **481** | **962** |
|  |  | **100%** | **100%** | **100%** |
| Severity  (first 1-2 weeks) | I was hospitalised | 30 | 16 | 46 |
|  |  | 6.2% | 3.3% | 4.8% |
|  | I experienced symptoms and had to rest in bed | 363 | 381 | 744 |
|  |  | 75.5% | 79.2% | 77.3% |
|  | I experienced symptoms but was able to carry on with daily activities | 79 | 71 | 150 |
|  |  | 16.4% | 14.8% | 15.6% |
|  | I was asymptomatic | 9 | 13 | 22 |
|  |  | 1.9% | 2.7% | 2.3% |
|  | **Total** | **481** | **481** | **962** |
|  |  | **100%** | **100%** | **100%** |
| Symptoms | My symptoms were worse at the beginning (the first 1-2 weeks) and then got better | 23 | 179 | 202 |
|  |  | 4.8% | 37.6% | 21.2% |
|  | My symptoms were worse at the beginning (the first 1-2 weeks) and then mostly got better but some lingered | 152 | 167 | 319 |
|  |  | 32.0% | 35.1% | 33.5% |
|  | After the first 1-2 weeks, my symptoms got better but then the same symptoms kept coming back | 67 | 21 | 88 |
|  |  | 14.1% | 4.4% | 9.3% |
|  | After the first 1-2 weeks, my symptoms got better but I then developed new symptoms | 60 | 15 | 75 |
|  |  | 12.6% | 3.2% | 7.9% |
|  | Most of my symptoms lasted for 2-3 weeks | 25 | 39 | 64 |
|  |  | 5.3% | 8.2% | 6.7% |
|  | Most of my symptoms lasted for 4-12 weeks | 62 | 23 | 85 |
|  |  | 13.1% | 4.8% | 8.9% |
|  | Most of my symptoms lasted for more than 12 weeks | 81 | 6 | 87 |
|  |  | 17.1% | 1.3% | 9.2% |
|  | I cannot answer this question (e.g. you had COVID very recently) | 5 | 26 | 31 |
|  |  | 1.1% | 5.5% | 3.3% |
|  | **Total** | **475** | **476** | **951** |
|  |  | **100%** | **100%** | **100%** |
