# Supplementary material for: Psychological consequences of long COVID: comparing trajectories of depressive and anxiety symptoms before and after contracting SARS-CoV-2 between matched long and short COVID groups
Source: Br J Psychiatry. Author manuscript; Available in PMC 2023 Feb 1. (PMC7614126; doi:10.1192/bjp.2022.155)
Supplement: Supplementary file 1 [file EMS155874-supplement-Supplementary_Material.pdf]

Supplementary Material

Model specification:

$$Y_{ij} = \pi_{0i} + \pi_{1i} time_{ij} \; + \; \pi_{2i} time_{ij}^{2} \; + \varepsilon_{ij} \qquad \text{(Eq. 1)}$$

$$\pi_{0i} = \gamma_{00} + \gamma_{01} group_i + \gamma_{01} month_i + \zeta_{0i} \qquad \text{(Eq. 2)}$$

$$\pi_{1i} = \gamma_{10} + \gamma_{11} group_i + \zeta_{1i} \qquad \text{(Eq. 3)}$$

$$\pi_{2i} = \gamma_{20} + \gamma_{21} group_i + \zeta_{2i} \qquad \text{(Eq. 4)}$$

The growth curve model is presented mathematically in Equation (1) to (4). Equation (1) addresses intra-individual growth where Y_{ij}, depressive or anxiety symptoms for the individual i at time j, is a function of time and its quadratic term. π_{0i} represents the individual i's initial status. π_{1i} and π_{2i} represent growth rates and ε_{ij} is the residual term. Equation (2) to (4) addresses inter-individual differences in the initial status (intercept) and growth rates. γ_{00}, $\gamma_{10}$and γ_{20} represent the population average initial status (intercept) and growth rates. ζ_{0i}, ζ_{1i} and ζ_{2i} are parameter residuals.

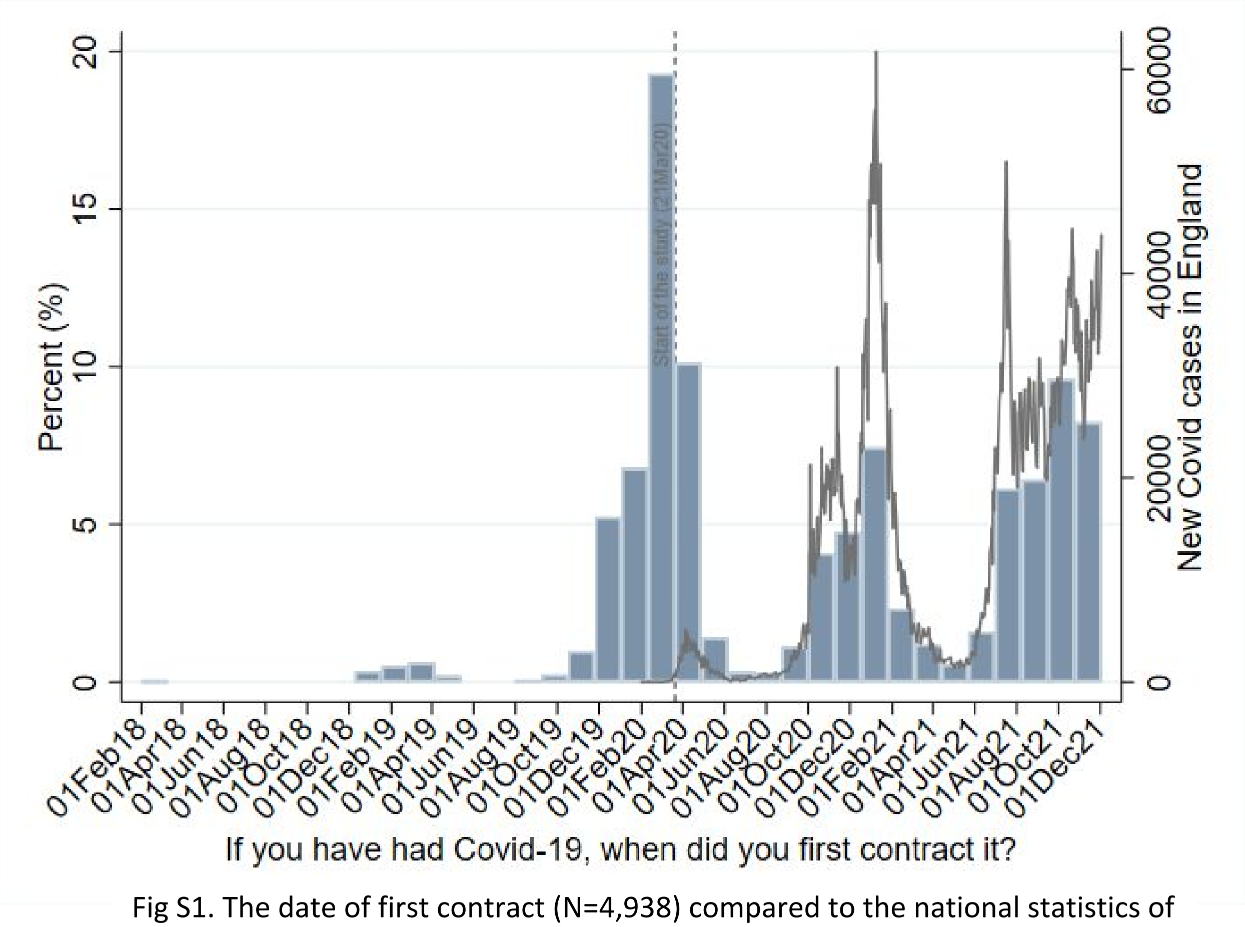

Fig S1. The date of first contract (N=4,938) compared to the national statistics of COVID-19 cases in England

Sources: COVID-19 Social Study, https://coronavirus.data.gov.uk/

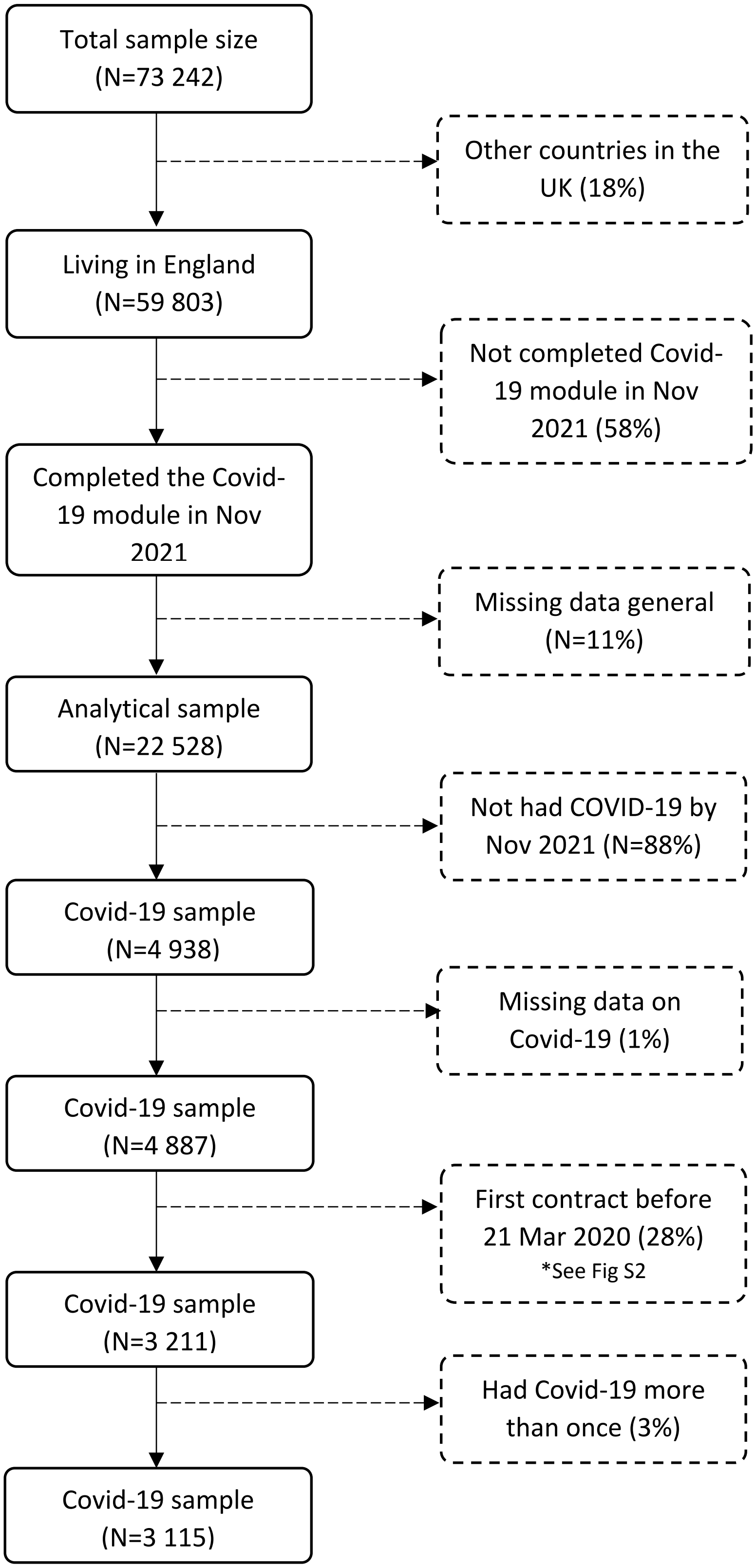

Fig S2. Sample selection process

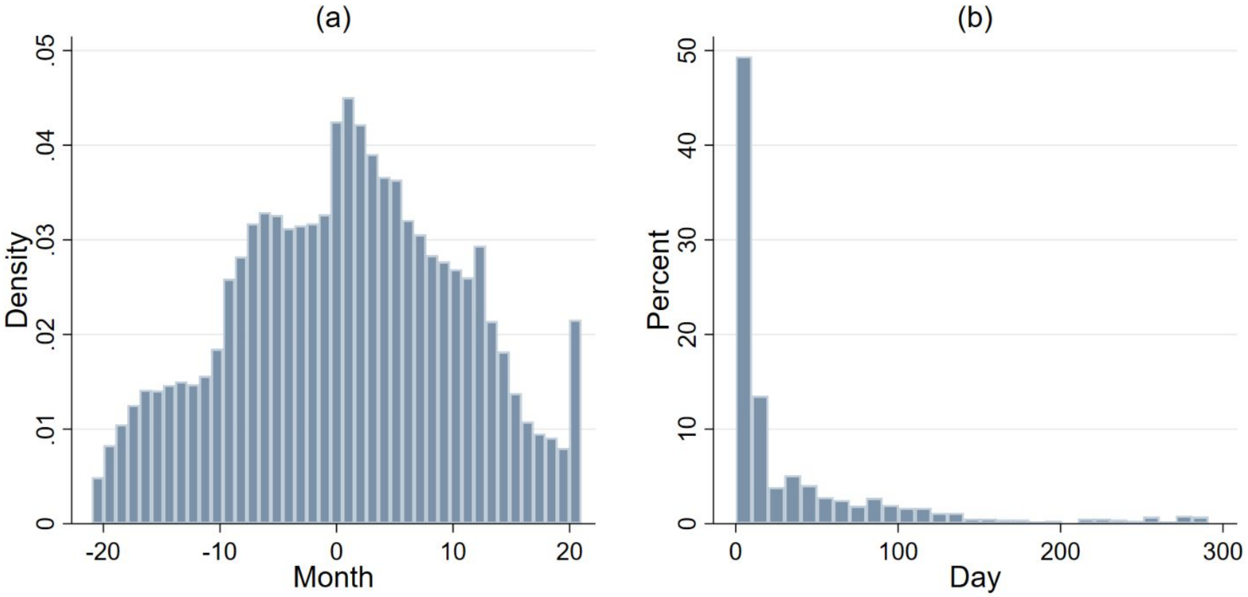

Fig S3. (a) Distribution of survey months since contracting COVID-19 in the one-to-one matched sample (total number of observations=13,325), (b) Distribution of the gap (day) between self-reported COVID-19 date and the closest survey date (either before or after, number of participants=962)

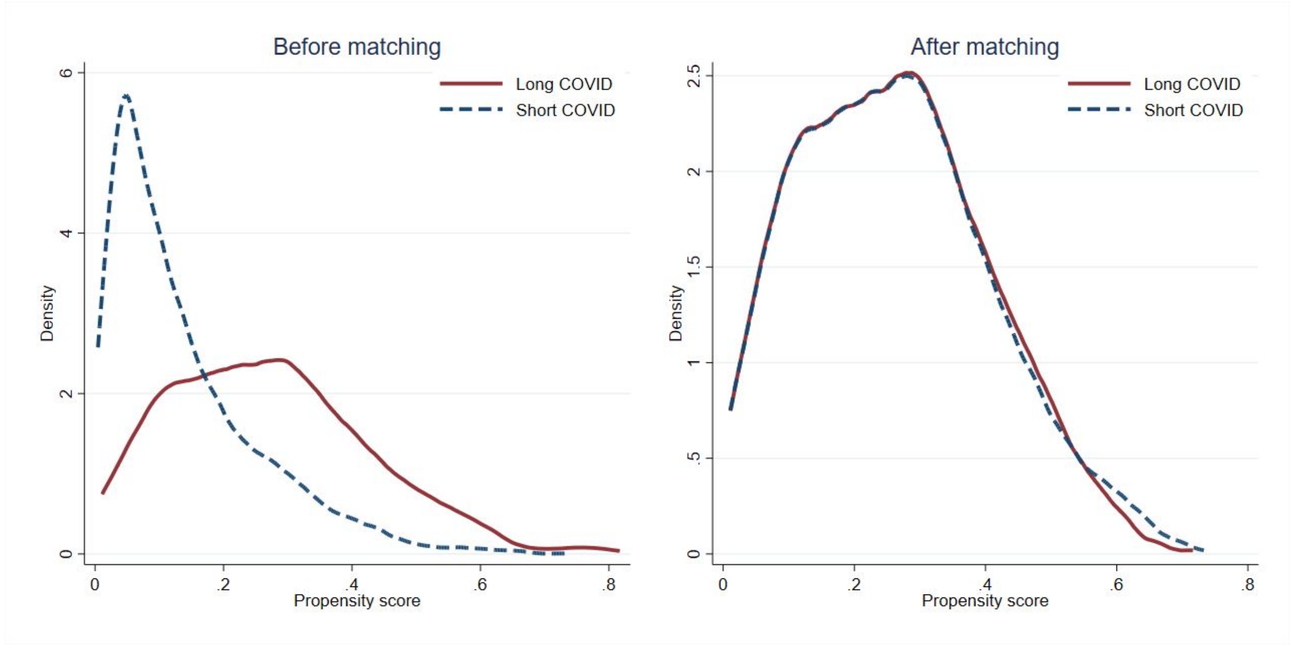

Fig S4. Propensity score density before and after matching

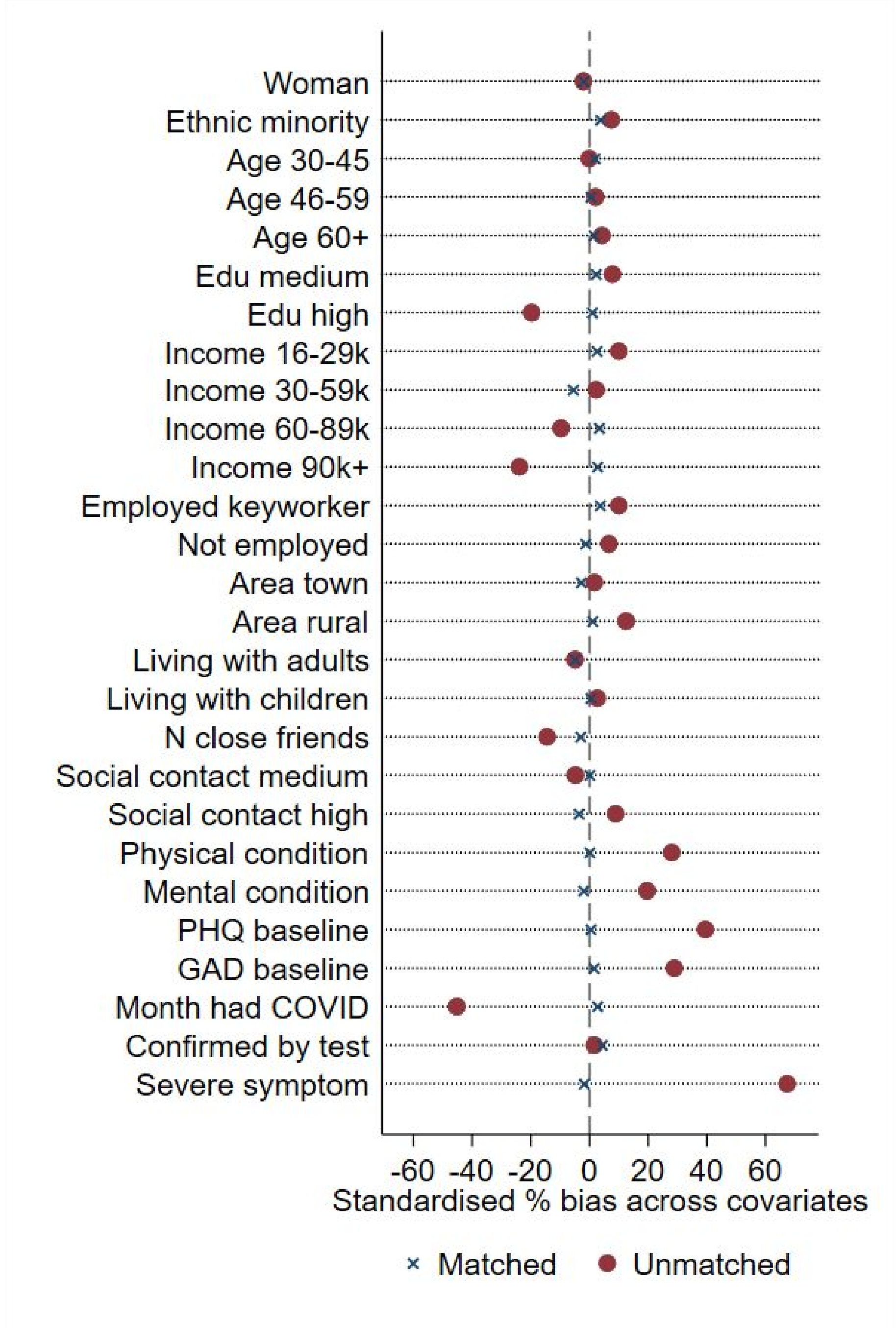

Fig S5. Standardised percentage bias for each covariate before and after matching

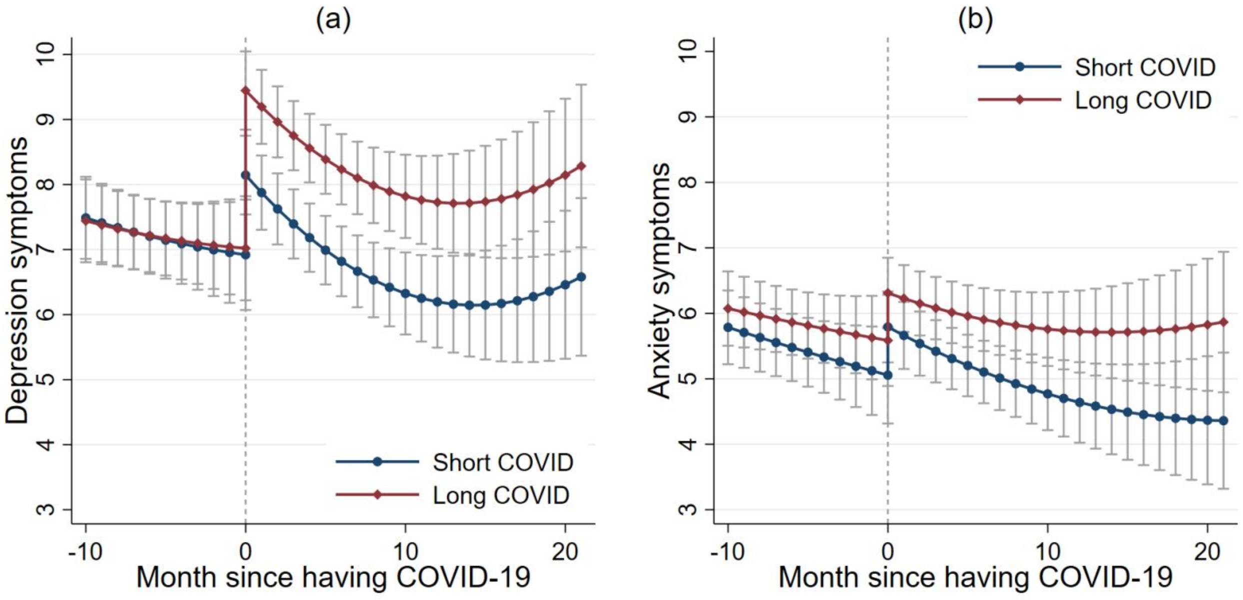

Fig S6. Predicted growth trajectories by Covid-19 group with 95% confidence intervals

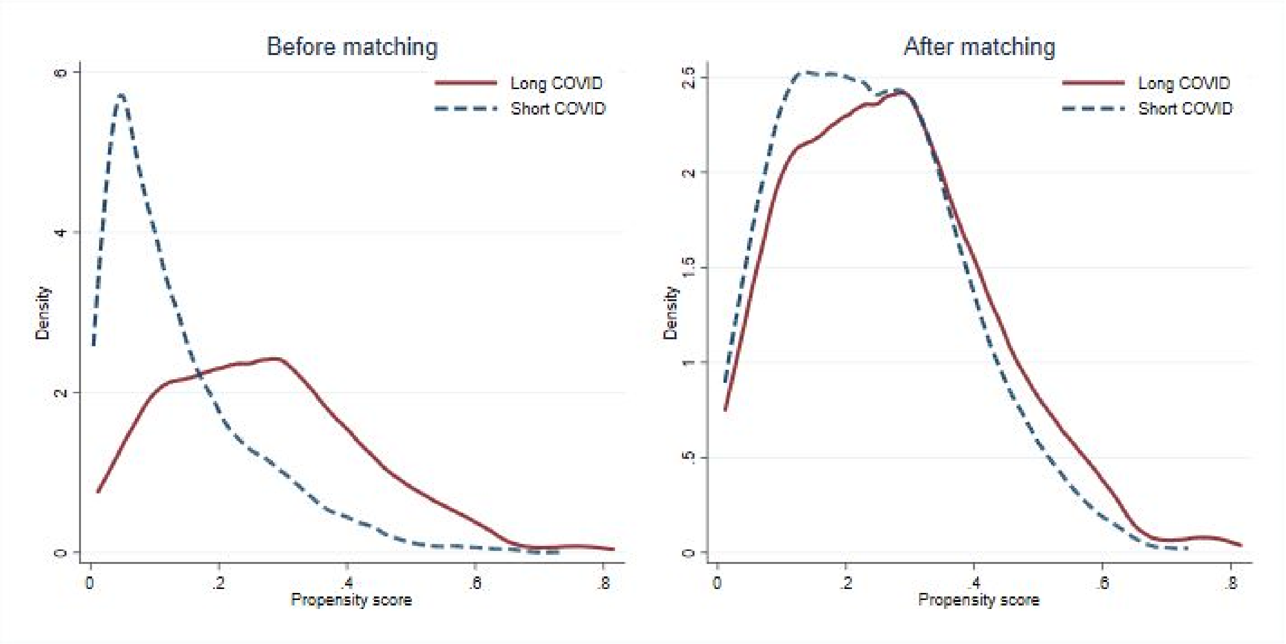

Fig S7. Propensity score density before and after matching based on one-to-many nearest neighbour matching

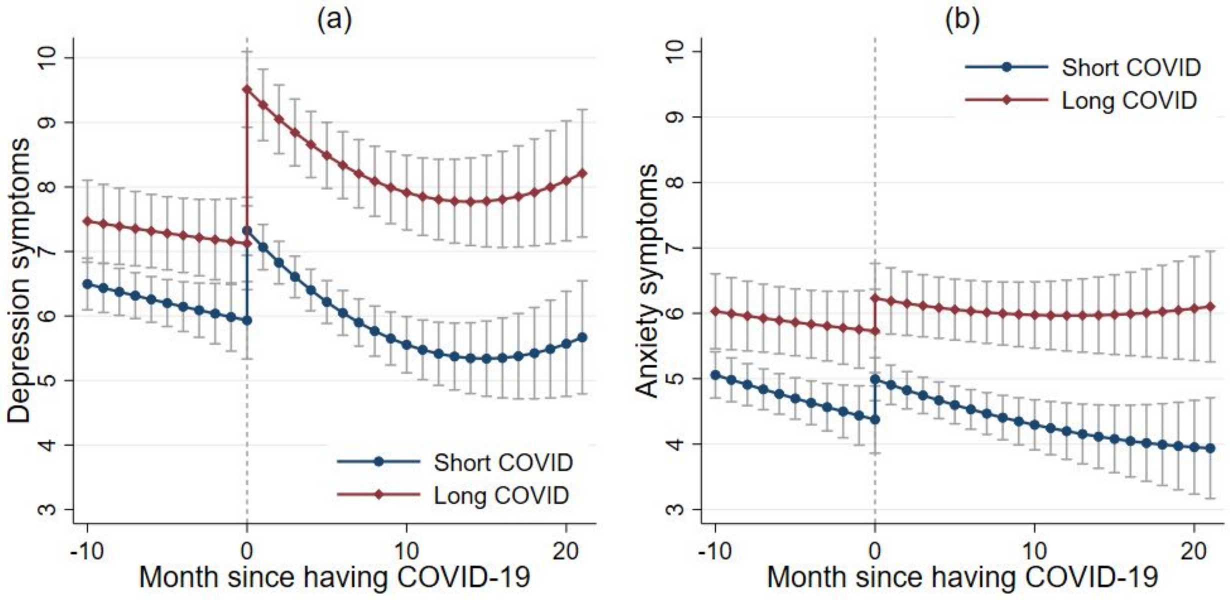

Fig S8. Predicted growth trajectories by Covid-19 group with 95% confidence intervals based on one-to-many nearest neighbour matching

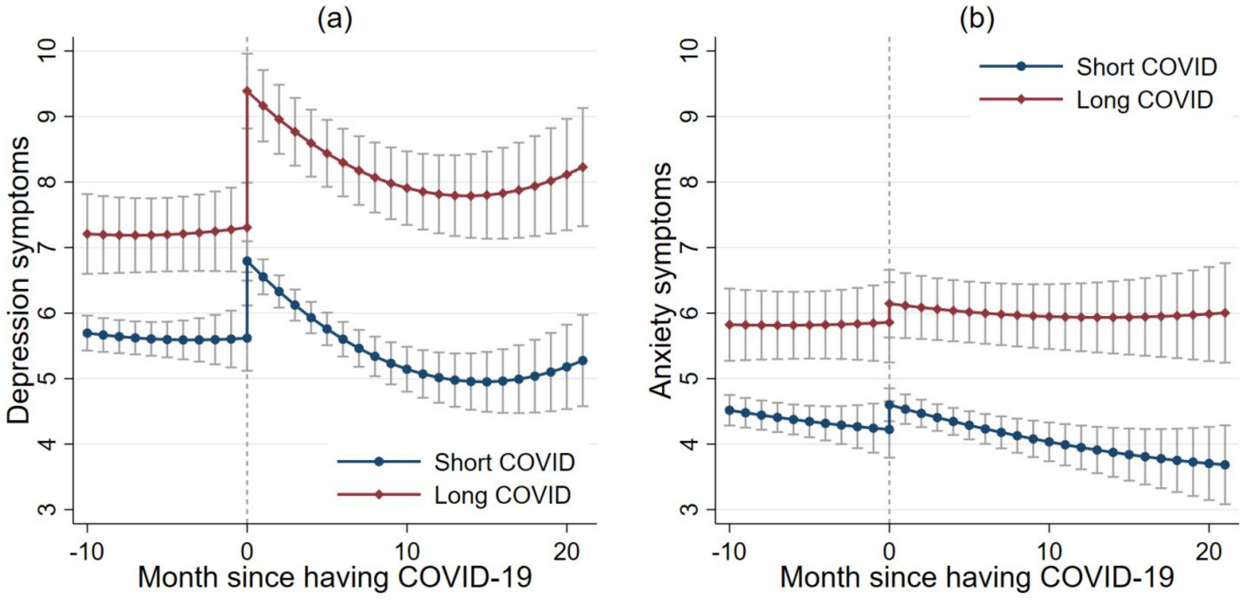

Fig S9. Predicted growth trajectories by Covid-19 group with 95% confidence intervals based on kernel matching *(NB these sensitivity analyses yielded less balanced matches so apparent differences in pre-infection mental health should be interpreted with caution)*

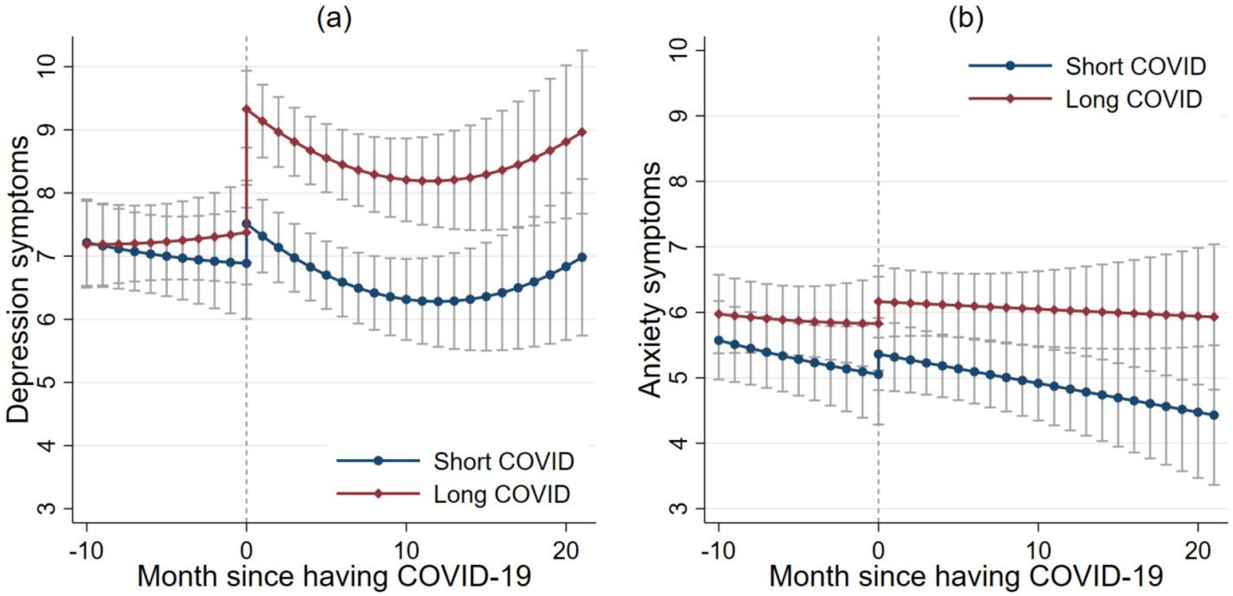

Fig S10. Predicted growth trajectories by Covid-19 group with 95% confidence intervals based on one-to-one matching excluding cases based on symptoms

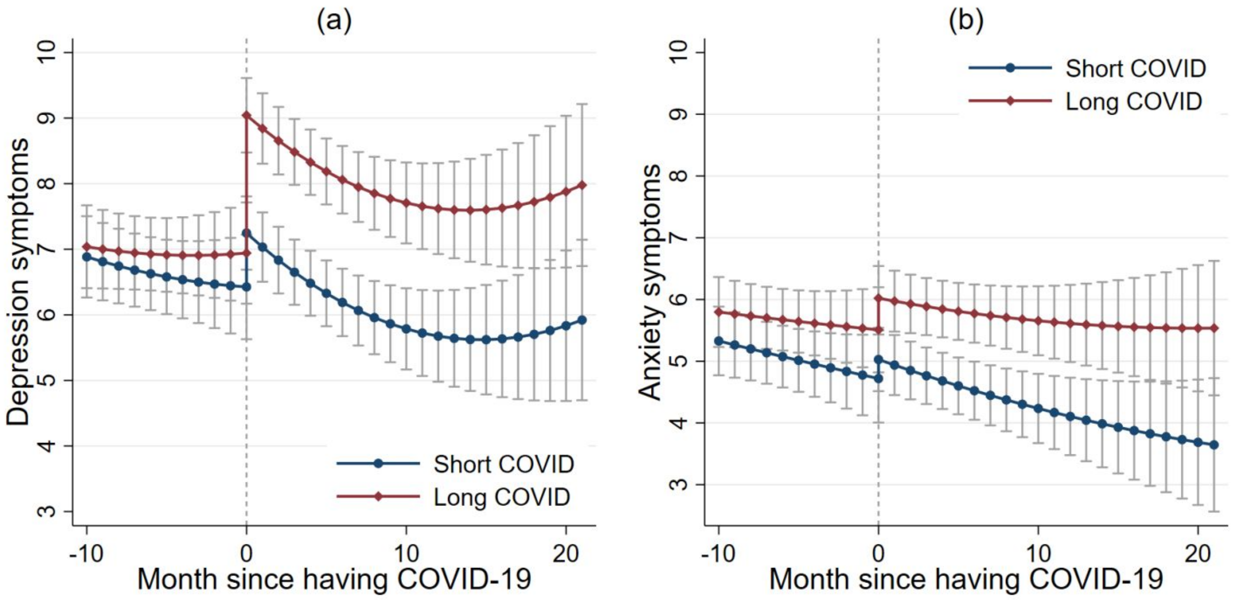

Fig S11. Predicted growth trajectories by Covid-19 group with 95% confidence intervals based on one-to-one matching excluding people who were unsure about their long Covid diagnosis

Table S1 Results from Propensity Score Matching (one-to-one matching without replacement)

	Coef.	SE	95% CI	p
Women (vs men)	-0.33	0.13	[-0.60--0.07]	0.013
Ethnic minority (vs white)	0.30	0.23	[-0.16-0.76]	0.197
Age 30-45 (vs 18-29)	0.48	0.24	[0.01-0.95]	0.046
Age 46-59 (vs 18-29)	0.45	0.24	[-0.01-0.91]	0.056
Age 60+ (vs 18-29)	0.64	0.26	[0.13-1.14]	0.013
Education: A levels or equivalent (vs GCSE or below)	-0.23	0.19	[-0.60-0.13]	0.208
Education: Degree or above (vs GCSE or below)	-0.40	0.16	[-0.72--0.09]	0.013
Income: ≤£16,000-29,999 (vs <£16,000)	-0.18	0.19	[-0.55-0.19]	0.334
Income: ≤£30,000-59,999 (vs <£16,000)	-0.25	0.19	[-0.61-0.12]	0.187
Income: ≤£60,000-89,999 (vs <£16,000)	-0.48	0.22	[-0.91--0.05]	0.029
Income: ≥£90,000 (vs <£16,000)	-0.93	0.26	[-1.43--0.42]	<0.001
Employed, non-keyworker (vs Employed, keyworker)	0.14	0.13	[-0.11-0.39]	0.282
Not employed (vs Employed, keyworker)	-0.03	0.15	[-0.32-0.27]	0.864
Area of living: Town (vs City)	0.09	0.12	[-0.15-0.34]	0.452
Area of living: Rural (vs City)	0.51	0.15	[0.21-0.81]	0.001
Living with adult only (vs Living alone)	0.13	0.17	[-0.2-0.46]	0.444
Living with children (vs Living alone)	0.39	0.18	[0.03-0.74]	0.032
Number of close friends	-0.01	0.02	[-0.05-0.03]	0.655
Social contact: Once or twice a week (vs Twice a month or less)	-0.08	0.13	[-0.33-0.18]	0.553
Social contact: Three times a week or more (vs Twice a month or less)	-0.05	0.14	[-0.33-0.23]	0.733
Physical health condition: Yes (vs No)	0.32	0.11	[0.10-0.54]	0.004
Mental health condition Yes (vs No)	0.01	0.15	[-0.28-0.29]	0.955
Baseline depressive symptoms	0.05	0.02	[0.02-0.08]	0.002
Baseline anxiety symptoms	0.00	0.02	[-0.03-0.04]	0.885
Month had COVID-19	-0.09	0.01	[-0.11--0.07]	<0.001
Confirmed by testing (COVID-19 or antibody test): Yes (vs No)	0.69	0.14	[0.41-0.98]	<0.001
Severe symptoms (hospitalised or had to stay in bed): Yes (vs No)	1.37	0.13	[1.12-1.63]	<0.001
Constant	-2.47	0.41	[-3.28--1.66]	<0.001

Table S2 Sample characteristics by matched Covid-19 group

		Long Covid (N=481)	Short Covid (N=481)
Gender	Men	21.8%	21.0%
	Women	78.2%	79.0%
Ethnicity	White	94.2%	95.0%
	Ethnic minority	5.8%	5.0%
Age group	18-29	6.0%	7.7%
	30-45	31.6%	30.8%
	46-59	39.1%	38.9%
	60+	23.3%	22.7%
Education	GCSE or below	15.4%	16.6%
	A levels or equivalent	19.5%	18.7%
	Degree or above	65.1%	64.7%
Annual income	<£16,000	14.3%	14.8%
	≤£16,000-29,999	23.7%	22.7%
	≤£30,000-59,999	38.3%	41.0%
	≤£60,000-89,999	16.2%	15.0%
	≥£90,000	7.5%	6.7%
Employment status	Employed, keyworker	39.1%	40.1%
	Employed, non-keyworker	32.6%	31.0%
	Not employed	28.3%	28.9%
Area of living	City	32.0%	31.0%
	Town	46.8%	48.2%
	Rural	21.2%	20.8%
Living status	Living alone	14.8%	12.5%
	Living with adult only	48.2%	50.7%
	Living with children	37.0%	36.8%
Social contact	Twice a month or less	35.3%	33.7%
	Once or twice a week	33.3%	33.3%
	Three times a week or more	31.4%	33.1%
Number of close friends		4.7 (3.1)	4.8 (3.1)
Physical health condition	Yes	44.1%	44.1%
	No	55.9%	55.9%
Mental health condition	Yes	23.1%	23.9%
	No	76.9%	76.1%
Baseline depressive symptoms		8.3 (5.9)	8.2 (6.0)
Baseline anxiety symptoms		6.5 (5.4)	6.4 (5.2)
Month had COVID-19		10.5 (6.5)	10.3 (7.1)
Confirmed by testing (COVID-19 or antibody test)	Yes	74.6%	72.8%
	No	25.4%	27.2%
Severe symptoms (hospitalised or had to stay in bed)	Yes	81.7%	82.5%
	No	18.3%	17.5%

Table S3. Detailed Covid experience measures by matched Covid-19 group

		Long Covid	Short Covid	Total
Test	Yes, a medical professional has formally diagnosed me with long Covid	112<br>23.3%	--	112<br>11.6%
	Yes, I have not been formally diagnosed but consider myself to have Long Covid	369<br>76.7%	--	369<br>38.4%
	No, I do not consider myself to have Long Covid	--	369<br>76.7%	369<br>38.4%
	I am unsure	--	112<br>23.3%	112<br>11.6%
	**Total**	**481**<br>**100%**	**481**<br>**100%**	**962**<br>**100%**
Severity (first 1-2 weeks)	I was hospitalised	30<br>6.2%	16<br>3.3%	46<br>4.8%
	I experienced symptoms and had to rest in bed	363<br>75.5%	381<br>79.2%	744<br>77.3%
	I experienced symptoms but was able to carry on with daily activities	79<br>16.4%	71<br>14.8%	150<br>15.6%
	I was asymptomatic	9<br>1.9%	13<br>2.7%	22<br>2.3%
	**Total**	**481**<br>**100%**	**481**<br>**100%**	**962**<br>**100%**
Symptoms	My symptoms were worse at the beginning (the first 1-2 weeks) and then got better	23<br>4.8%	179<br>37.6%	202<br>21.2%
	My symptoms were worse at the beginning (the first 1-2 weeks) and then mostly got better but some lingered	152<br>32.0%	167<br>35.1%	319<br>33.5%
	After the first 1-2 weeks, my symptoms got better but then the same symptoms kept coming back	67<br>14.1%	21<br>4.4%	88<br>9.3%
	After the first 1-2 weeks, my symptoms got better but I then developed new symptoms	60<br>12.6%	15<br>3.2%	75<br>7.9%
	Most of my symptoms lasted for 2-3 weeks	25<br>5.3%	39<br>8.2%	64<br>6.7%
	Most of my symptoms lasted for 4-12 weeks	62<br>13.1%	23<br>4.8%	85<br>8.9%
	Most of my symptoms lasted for more than 12 weeks	81<br>17.1%	6<br>1.3%	87<br>9.2%
	I cannot answer this question (e.g. you had COVID very recently)	5<br>1.1%	26<br>5.5%	31<br>3.3%
	**Total**	**475**<br>**100%**	**476**<br>**100%**	**951**<br>**100%**
